# Comprehensive characterization of TNFSF14/LIGHT with implications in prognosis and immunotherapy of human gliomas

**DOI:** 10.3389/fimmu.2022.1025286

**Published:** 2022-10-20

**Authors:** Mingzhi Han, Yanfei Sun, Wenbo Zhao, Guo Xiang, Xu Wang, Zheng Jiang, Zhiwei Xue, Wei Zhou

**Affiliations:** ^1^ Department of Neurosurgery, Qilu Hospital, Cheeloo College of Medicine and Institute of Brain and Brain-Inspired Science, Shandong University, Jinan, China; ^2^ Jinan Microecological Biomedicine Shandong Laboratory, Jinan, China; ^3^ Shandong Key Laboratory of Brain Function Remodeling, Jinan, China; ^4^ Medical Integration and Practice Center, Cheeloo College of Medicine, Shandong University, Jinan, China; ^5^ Department of Radiation Oncology, Qilu Hospital, Shandong University, Jinan, China

**Keywords:** glioma, LIGHT, immune response, prognosis, tumor microenvironment

## Abstract

Glioblastoma multiforme (GBM) is a common central neural system malignant tumor among adults. Alongside its microscopic spread, immunosuppression in the tumor microenvironment also induces its refractoriness, which makes immunotherapy for GBM particularly important. Unfortunately, traditional immune checkpoint inhibitors (ICIs) often show limited therapeutic effects in GBM clinical trials, and new therapeutic strategies or targets are urgently needed. TNFSF14/LIGHT is a novel immune checkpoint molecule that plays essential roles in both innate and acquired immunity. Despite recent advances in our understanding of the function of TNFSF14/LIGHT in a variety of cancer types, the clinical and immunological importance of TNFSF14/LIGHT in human gliomas has not been fully explained. Here, we employed a comprehensive in silico analysis with publicly available data to analyze the molecular and immune characteristics of TNFSF14/LIGHT to explore its feasibility as an immunotherapy target. Totally, 2215 glioma cases were enrolled in the current study. Immunohistochemistry staining based on patient tissues (n = 34) was performed for the validation. TNFSF14/LIGHT was expressed higher in higher-WHO-grade gliomas and mesenchymal subtypes, and it was sensitive as a prognostic marker in GBM and low-grade glioma (LGG). A nomogram prognostic model was established based on TNFSF14/LIGHT expression together with other risk factors. Additionally, Gene Ontology and pathway analysis revealed that TNFSF14/LIGHT participated in T-cell activities and inflammatory processes. Moreover, analysis based on the structure and interactions of TNFSF14/LIGHT revealed its mutation sites in tumors as well as crucial interacting proteins. Analysis of IMvigor210 indicated the role of TNFSF14/LIGHT in immunotherapy. Altogether, our results reveal an underlying role of TNFSF14/LIGHT as an immunotherapy target in GBM.

## Introduction

In adults, glioblastoma multiforme (GBM) is the most frequent malignant primary brain tumor. Even after aggressive surgery followed by radiotherapy and chemotherapy, patients show a median overall survival (OS) of no more than 15 months beyond the primary diagnosis, and their 5-year survival rate is less than 5% (1, 2). The dismal prognosis can be attributed to the infiltration and microscopic spread of GBM cells (3, 4). Recurrence is almost inevitable, which contributes to the difficulty in curing this refractory disease.

Tremendous progress in molecular and histological research has also uncovered some underlying mechanisms that might explain its refractory features. Like other tumors, GBM features a high vascularization rate and facilitates the formation of abnormal vessels, which results in special tumor vessels with tortuous and dilated structures, enhanced permeability, and low pericyte coverage (5, 6). The abnormal structure and hyperpermeability of the tumor vessels induce aberrations in fluid dynamics and oxygen supply, which can provoke the growth of GBM or other cancer cells and reduce the effect of cytotoxic drugs (5, 7). Concerning the immune signature, although confined to the intracranial compartment with rare extracranial metastasis, GBM is a notorious immune evader characterized by a severely immunosuppressive tumor microenvironment, including little T-cell infiltration (8, 9). The immunosuppression status inside GBM is demonstrated by upregulation of PD-L1 in almost 50% of primarily diagnosed and 45% of recurrent GBM cases (9), and PD-L1 expression is negatively correlated with prognosis (10, 11). In addition, unlike other malignancies, GBM manifests attenuated T-cell infiltration but increased macrophage infiltration (12). It has been reported that increased tumor-infiltrating CD8+ T cells could indicate prolonged survival in GBM patients (13), and the higher grade of glioma is parallel to increased regulatory T cell (Treg) infiltration (14).

Cancer immunotherapy has been developed to destroy cancer cells by harnessing the antitumor immune system. Among the versatile effects of antitumor immunity, immune checkpoints are gradually attracting the attention of researchers, and a body of evidence has indicated their importance. Our previous study suggested that one of the immune checkpoints named herpes virus entry mediator (HVEM) could be a prognostic marker associated with OS and could be a target for immune checkpoint blockade therapy (15). One of its ligands, LIGHT, otherwise known as tumor necrosis factor superfamily member 14 (TNFSF14) or CD258, could improve the effect of immunotherapy in multiple cancer models, such as lung carcinoma (16), breast carcinoma (17), cervical cancer (18), prostate cancer (19) and GBM (20). LIGHT can exert its functions as both a soluble and a surface-bound membrane protein (21). Only its homotrimeric form interacts with its two primary receptors, HVEM and lymphotoxin-β receptor (LTβR) (22, 23). In cell signaling, it has been reported that LIGHT has four distinct functions: activating 1) LTβR and 2) HVEM, 3) disrupting the HVEM-BTLA complex in surface-bound form, and 4) facilitating HVEM-BTLA complex formation in the soluble form (24). In tumor immunology, LIGHT can stimulate NK cells to produce IFNγ *via* nuclear factor-κB (NFκB) RelA/p50 signaling (25, 26). The LIGHT produced by NK cells plays an essential role in NK-DC crosstalk to enhance antitumor activity (27). In addition, intratumoral expression of LIGHT can drastically raise the number of dendritic cells (DCs) in situ, possibly exerting positive effects on antitumor immunity (28). Furthermore, it has been reported that LIGHT can sustain the function of CD8+ effector T cells (29), as demonstrated by the fact that CD8+ T-cell activation and CTL activity are suppressed in LIGHT-deficient mice (30). In addition to the effects mentioned above for immunotherapy, previous studies indicate LIGHT’s positive effect on the normalization of vasculature and formation of high endothelial venules (HEVs), and the generation of tertiary lymphoid structures (TLS) is promoted as a consequence of HEV formation (31–33). The delivery of LIGHT has been achieved *via* carriers such as adenovirus and fusion protein (21). These studies suggest a promising function of LIGHT in immunotherapy.

Recent studies showed that LIGHT was also upregulated in more aggressive more gliomas, taking part in the immune function of macrophages, T cells, and APCs. LIGHT functions as a biomarker to identify immunologic subtypes and prognosis with other ICGs in GBM (34) (35). Unfortunately, both studies had limited sample sizes. The confirmation processes were mainly based on bioinformatics analysis without experimental verification. Additionally, the studies were focused on themolecular aspects, and there was no analysis at the structural level. These restrictions limit the development of relevant inhibitors.

Here, we further validated the function of LIGHT in 2215 cases of glioma of different grades. In an adequate sample size, we investigated the potential roles of LIGHT in antitumor immunity. We utilized single-cell sequencing data and collected clinical tissues (n=34) to verify the conclusions. We explored the effects of different genetic alterations and LIGHT-binding proteins, which could help researchers investigate the potential molecular mechanism of LIGHT in the pathogenesis or clinical prognosis of LGG and GBM. Our findings demonstrate the significance of LIGHT in the malignancy of gliomas in a large sample and provide insight into the role of LIGHT in glioma immune infiltration and its correlation with immune checkpoints. Analysis based on the structure and interactions of LIGHT reveals its mutation sites in tumors as well as its crucial interacting proteins, providing a new perspective for future study. Immunotherapy response analysis based on the IMvigor210 cohort indicated LIGHT inhibition as a synergistic immunotherapeutic approach.

## Materials and methods

### Ethics statement

All the protocols in our study were admitted by the Research Ethics Committee of Shandong University and the Ethics Committee of Qilu Hospital (Shandong, China) (SDULCLL2021-2-26). All experiments and analyses were performed under the guidance of corresponding protocols or guidelines and written informed consent was acquired from all patients.

### Clinical specimens

Glioma tissue specimens of WHO grade II-IV were collected by the Department of Neurosurgery, Qilu Hospital of Shandong University, from patients (n = 29) who underwent surgery, and the specimens were embedded in paraffin. Normal brain tissue specimens were collected from patients with head injuries (n = 5) who had to undergo partial brain resection for decompression. Genomic information and related clinical details for samples in The Cancer Genome Atlas Research Network (n = 667; TCGA, http://cancergenome.nih.gov), Rembrandt (n = 510; http://rembrandt.nci.nih.gov), CGGA (n = 693; http://www.cgga.org.cn), and the Gene Expression Omnibus (https://www.ncbi.nlm.nih.gov/geo/) were used for analysis. Clinical data and RNA sequence information for the IMvigor210 group were obtained from http://research-pub.gene.com/IMvigor210CoreBiologies/packageVersions/.

### Immunohistochemistry

The specimens were sliced into 4 μm sections, covered with paraffin, and stored at 4°C until immunohistochemistry was performed. The immunohistochemistry procedure was based on our previous study (15) with slight modifications. The immunohistochemistry kit was obtained from Zhongshan Golden Bridge Biotechnology, Beijing, Co., Ltd., and included 3% H2O2, normal goat serum for blocking streptavidin marked with horseradish peroxidase, and diaminobenzidine reaction agent. The sodium citrate antigen retrieval solution was acquired from Beijing Solarbio Science & Technology Co., Ltd. The polyclonal antibody against LIGHT was acquired from Sigma-Aldrich (HPA012700) and was applied at a concentration of 1:50.

### Biological function and gene set enrichment analysis

RStudio (version 3.6.1, https://rstudio.com/) was utilized to analyze the correlation between the expression of LIGHT and other genes. The LIGHT-associated KEGG pathway analysis and biological process identification were accomplished by the DAVID online tool (https://david.ncifcrf.gov/) with a threshold of P < 0.01. The hallmark gene sets were downloaded from the Molecular Signatures Database (MSigDB), and the correlations between LIGHT and the gene sets were analyzed with GSEA software (version 4.0.3, https://www.gsea-msigdb.org/gsea/index.jsp). The somatic mutation and somatic copy number alteration (CNA) data were downloaded from the TCGA database. GISTIC (version 2.0, http://software.broadinstitute.org/software/igv/GISTIC) was applied to analyze the CNA data.

### Genetic alteration analysis

After login into the cBioPortal website (https://www.cbioportal.org), we selected “TCGA Pan-Cancer Atlas Studies” in the “Quickselect” area and typed “TNFSF14” for inquiries regarding the genetic alteration traits of LIGHT/TNFSF14. The “Cancer Types Summary” module contained the results of the alteration frequency, mutation type, and CNA (copy number alteration) across LGG and GBM samples. The “Mutations” module enables the presentation of the TNFSF14 mutation site information in the protein structure schematic or the three-dimensional structure. In order to learn more about the variations in overall survival across TCGA cancer patients with and without a genetic mutation of LIGHT/TNFSF14, we also employed the “Comparison” module. There were also generated Kaplan-Meier graphs with log-rank P values.

### LIGHT/TNFSF14-related gene enrichment analysis

The STRING website (https://string-db.org) was first searched using the query “TNFSF14” and the organism “*Homo sapiens*” Then, we established the following key criteria: the minimum necessary interaction score [“Low confidence (0.150)”, the significance of network edges (“evidence”), the maximum number of interactors to display (“no more than 50 interactors” in the first shell), and the active interaction sources (“experiments”). Finally, the LIGHT-binding proteins that have been experimentally determined were retrieved.

Based on datasets of all TCGA cancers and normal tissues, we used the “Similar Gene Detection” module of GEPIA2 to identify the top 1000 LIGHT-correlated target genes. We also carried out a pairwise Pearson correlation study of LIGHT and particular gene expression using the “correlation analysis” module of GEPIA2. For the dot plot, the log2 TPM was used. The correlation coefficient (R) and the P value are shown. Additionally, we used TIMER2’s “Gene Corr” module to provide the heatmap data of the chosen genes, which includes the partial correlation (cor) and P value in the purity-adjusted Spearman’s rank correlation test.

We used Hiplot (https://hiplot.com.cn/) to conduct an intersection analysis to compare the LIGHT-binding and interacting genes. Moreover, we performed KEGG (Kyoto Encyclopedia of Genes and Genomes) pathway analysis and GO (Gene Ontology) enrichment analysis of LIGHT-binding proteins and identified them with the “ggplot2” (https://cran.r-project.org/web/packages/ggplot2/index.html) R package.

### Statistical analysis

The distribution of data was examined by the Kolmogorov−Smirnov test. Student’s T-test and one-way ANOVA with Bonferroni’s posthoc test was performed with GraphPad Prism (version 7.04, https://www.graphpad.com/; La Jolla, CA, USA). The survival analysis with Kaplan−Meier curves was compared using the log-rank test. The data are presented as the mean ± SEM. All tests were two-sided, and P < 0.05 was set as the threshold of statistical significance.

## Results

### Escalated LIGHT expression is observed in aggressive gliomas

**Figure 1:**
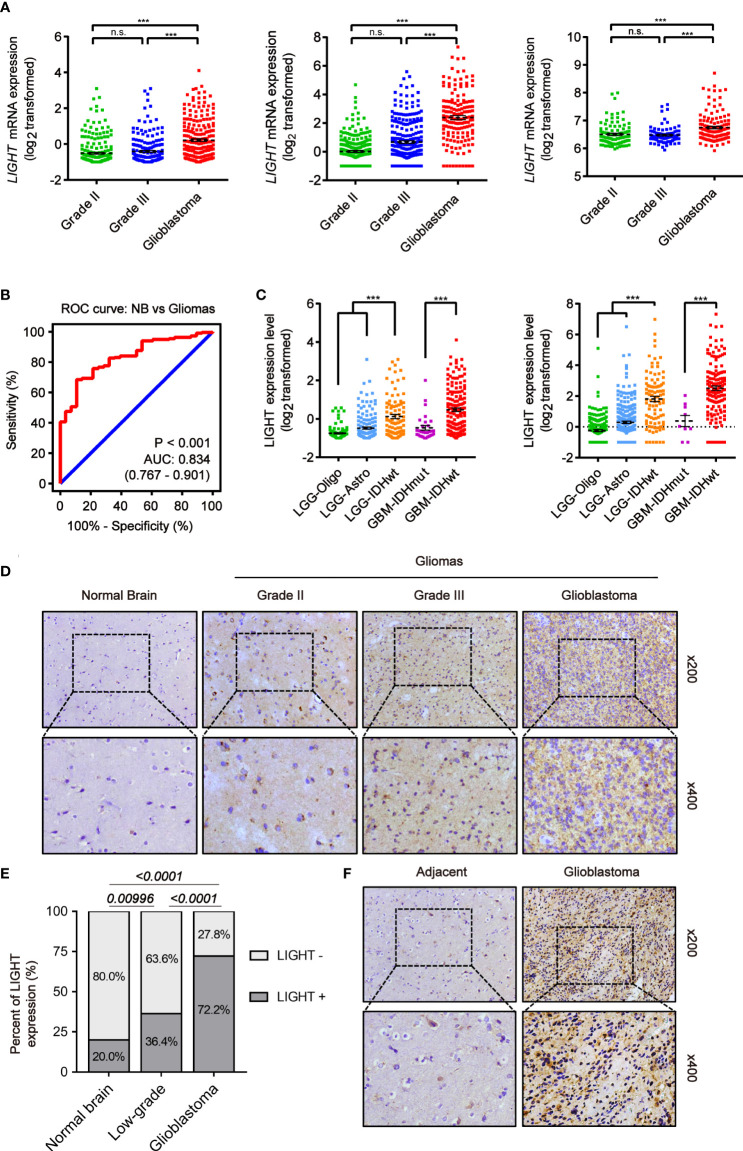
Increased LIGHT expression in higher-WHO-grade gliomas. **(A)** LIGHT levels of WHO categories II–IV mRNA expression in the TCGA (n = 667), CGGA (n = 693), and Rembrandt (n = 510). **(B)** The specificity and sensitivity of LIGHT were analyzed using a Receiver Operating Characteristic (ROC) curve, and it was proposed that LIGHT might be used as a diagnostic marker for gliomas. **(C)** The 2016 WHO classification was used to categorize the LIGHT mRNA expression levels in gliomas from the TCGA (n = 667) and CGGA (n = 693) datasets. **(D, F)** are representative immunohistochemistry images of healthy brain tissue, WHO grade II–IV gliomas (n = 29), and immunohistochemistry images of GBM with its surrounding tissue (n = 5). **(E)** Quantification of cases in which LIGHT is positive **(D)**. ns P >0.05; ***P < 0.001.

We analyzed the expression level of LIGHT in gliomas based on large-scale cohorts, including TCGA (n = 667), CGGA (n = 693) and Rembrandt (n = 510). The results suggested enhanced expression in GBM (WHO grade IV) compared with low-grade gliomas (LGG) (**Figure 1A**, P < 0.001). Furthermore, receiver operating characteristic (ROC) curve analysis concluded that gliomas and normal brain tissue could be distinguished by LIGHT expression (**Figure 1B**, area under the curve [AUC] value = 0.834, P < 0.001) Moreover, we analyzed its expression among different subtypes of gliomas in accordance with the 2016 WHO classifications of CNS tumors. In TCGA, the results showed repressed LIGHT expression in the LGG-Oligo subtype (IDHmut, 1p/19q codeletion) and LGG-Astro (IDHmut, 1p/19q non-codeletion) compared with the LGG-IDHwt group, while LIGHT expression in GBM-IDHwt was higher than that in the GBM-IDHmut group (**Figure 1C**). Similar results were seen in the CGGA data (**Figure 1C**).

We next conducted IHC staining to confirm LIGHT protein expression levels in gliomas (n = 29) and normal brain tissue (n = 5). We performed the IHC staining on the tissue samples of normal brain and low-grade glioma as well as glioblastoma from Qilu Hospital. The results showed that the positive rate of LIGHT expression was higher with increasing glioma grade (**Figures 1D, E**). IHC staining in one specific GBM sample (case #040) exhibited an obvious difference in LIGHT expression between glioblastoma and its adjacent tissue, in which GBM was marked with higher LIGHT expression while the adjacent tissue had a lower level than the former (**Figure 1F**). The results showed that high expression of LIGHT predicted malignant glioma and correlated with its aggressive condition.

### The intertumoral and intratumoral heterogeneity of LIGHT in gliomas

**Figure 2:**
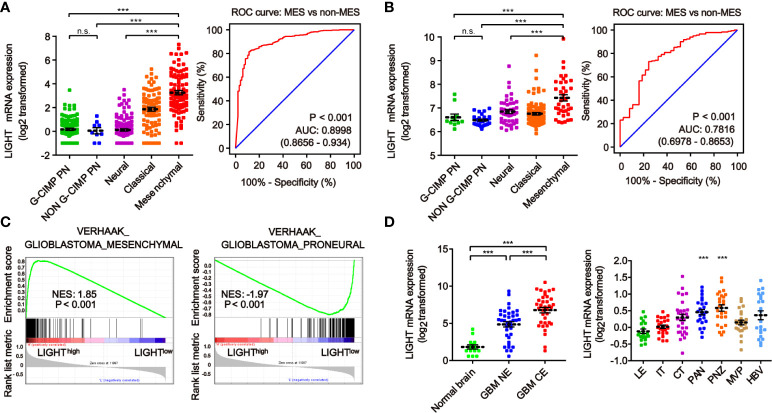
The heterogeneity of LIGHT expression within and between gliomas. The expression level of LIGHT mRNA in gliomas classified by the VERHAAK_2010 molecular classification, including CpG island methylator phenotype (G-CIMP) proneural, non-G-CIMP proneural, neural, classical, and mesenchymal subtypes, in the TCGA **(A)** and CGGA **(B)** databases. The corresponding ROC curves indicated that LIGHT is a diagnostic marker of the mesenchymal subtype. **(C)** GSEA enrichment of MES and PN signatures analyzing LIGHThigh vs LIGHTlow samples in the TCGA dataset of GBM. The normalized enrichment score (NES) and FDR are noted in the figure. **(D)** The analysis of LIGHT mRNA levels with the Gill dataset (n = 75) in different radiographical regions of GBM, including normal brain, T1 contrast-enhancing (CE) area and non-enhancing (NE; abnormal T2/FLAIR signal) area. **(E)** The analysis of LIGHT mRNA levels with IVY GBM RNA-seq data (n = 270) of different anatomical regions, including LE (Leading Edge), IT (Infiltrating Tumor), CT (Cellular Tumor), PAN (Pseudopalisading Cells Around Necrosis), PNZ (Perinecrotic Zone), MVP (Microvascular Proliferation), and HBV (Hyperplastic Blood Vessels). ns P >0.05; ***P < 0.001.

We next investigated the link between LIGHT expression and the molecular signatures of gliomas. The classifications of glioma based on molecular signatures have been commonly accepted as classical (CL), mesenchymal (MES), neural (NE) and proneural (PN), of which CL and MES are associated with aggressive behaviors and poor prognosis (36). We then interrogated the intratumor heterogeneity of LIGHT expression according to the VERHAAK_2010 classification scheme (37). The results from the TCGA dataset showed a higher LIGHT expression in the MES subtype than the NE, PN glioma CpG island methylator phenotype (G-CIMP) or non-G-CIMP subtypes (**Figure 2A**). The ROC curve further indicated a clear distinction of MES from non-MES (**Figure 2A**, AUC = 0.898, P < 0.001). Similar results were found in the CGGA dataset (**Figure 2B**). Gene set enrichment analysis indicated that higher-LIGHT-expression cases were enriched in the MES subtype, while lower LIGHT expression was mainly enriched in the PN subtype (**Figure 2C**).

According to the radiographical and anatomical characteristics of gliomas, we investigated the intratumoral heterogeneity of LIGHT expression. The T1 contrast-enhancing (CE) area, which represented GBM margins of edematous tissues with infiltrating tumor cells, exhibited higher LIGHT expression than the non-enhancing (NE; abnormal T2/FLAIR signal) area and normal brain (**Figure 2D**, P < 0.001, respectively). In addition, regarding the different anatomical areas, higher LIGHT expression was found within the Pseudopalisading Cells Around Necrosis (PAN) and Perinecrotic Zone (PNZ) areas than in other subareas (**Figure 2D**, P < 0.001, respectively). We then looked into the expression pattern of LIGHT according to the single-cell sequencing data. The results showed that GBM and immune cells were the major cell types that expressed LIGHT, and its expression was focused in the tumor core instead of the peripheral area (**Figure S1**).

### Elevated LIGHT expression can predict dismal prognosis of GBM

**Figure 3:**
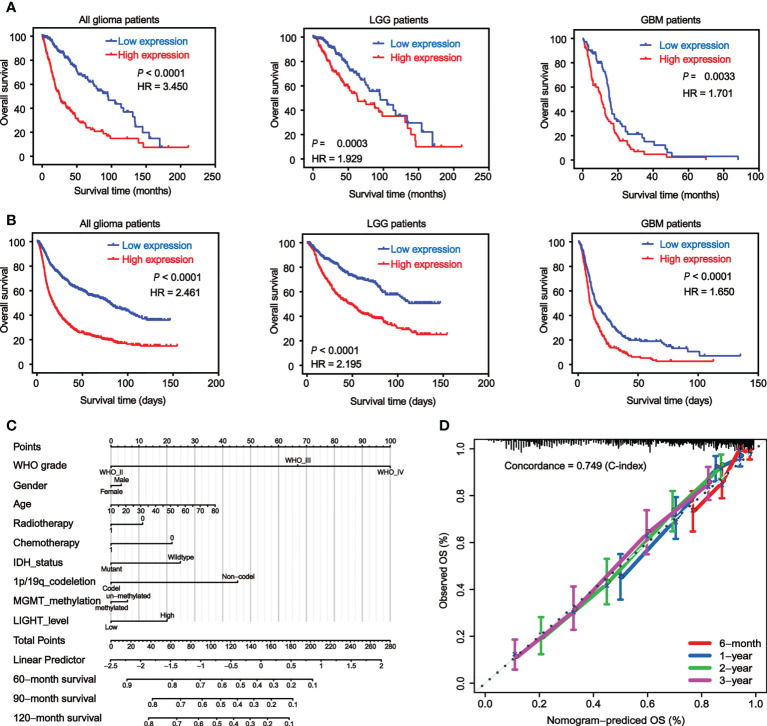
Elevated LIGHT expression predicts worse prognosis of GBM patients. Kaplan−Meier analysis of the overall survival (OS) rate of all glioma, LGG and GBM patients in the TCGA **(A)** and CGGA **(B)** datasets. **(C)** Nomogram predicting the proportion of glioma patients with OS. **(D)** Calibration plot showing the predicted and observed OS. The predictive performance of the model is compared to the perfect prediction, which is the diagonal line.

We used Kaplan-Meier analysis to determine the prognostic significance of LIGHT expression levels in gliomas. We analyzed the datasets from TCGA (**Figure 3A**) and CGGA (**Figure 3B**), and similar results were shown between these two databases. Among all glioma patients, high LIGHT expression predicted a shorter OS than low LIGHT expression (**Figures 3A, B**, P < 0.0001, respectively). More concretely, first, the analysis of LGG patient survival data led to the same conclusion: that higher LIGHT expression was associated with a more unfavorable prognosis in LGG patients (**Figures 3A, B**, P = 0.0003, P < 0.0001, respectively). Similar results were manifested in GBM patients (**Figures 3A, B**, P = 0.0033, P < 0.0001, respectively). We also found that progression-free survival (PFS) could be predicted in all glioma patients by LIGHT expression, where high LIGHT expression indicated a shorter PFS (**Figure S2A**, P < 0.0001), meaning high LIGHT expression might be associated with more rapid progression. This prediction value extended to LGG patients, as LGG patients with higher LIGHT expression exhibited shorter PFS (**Figure S2A**, P = 0.041), while higher or lower LIGHT expression in GBM patients did not make a statistically significant difference in PFS (**Figure S2A**, P = 0.455). However, in GBM patients with a molecular subtype of IDHwt, LIGHT expression was statistically prognostic, and higher LIGHT expression predicted a shorter OS (**Figure S2B**, P = 0.044).

Moreover, the prognostic value of LIGHT expression in the therapy response was also validated. According to the Kaplan-Meier curves, individuals with GBM who received radiotherapy or chemotherapy tended to have lower OS when their LIGHT expression was higher (**Figure S2C**, P = 0.046 and P = 0.008, respectively). Using univariate or multivariate Cox regression, we also investigated the potential of LIGHT expression as a standalone prognostic marker in gliomas. In the univariate Cox regression (**Table S1**, HR = 1.530, 95% CI = 1.437 to 1.629, P 0.001) and multivariate Cox regression (**Table S1**, HR = 1.107, 95% CI = 1.009 to 1.215, P = 0.031) using TCGA data, we came to the conclusion that LIGHT expression might be a potential independent prognostic marker for gliomas. Similar results were shown in the CGGA dataset with univariate Cox regression (**Table S1**, HR = 1.125, 95% CI = 1.089 to 1.161, P < 0.001) as well as multivariate Cox regression (**Table S1**, HR = 1.056, 95% CI = 1.012 to 1.102, P = 0.012).

Nomograms, which are visual computational systems of predictive statistical models, are frequently employed for cancer patients and have a proven benefit over conventional methods in terms of exact individual prediction. As a result, we used a nomogram model with integrated nomograms that contained clinical risk indicators like WHO grade, sex, and age (**Figure 3C**). Concordance [C-index] = 0.749 in calibration plots demonstrated that the nomograms performed well in comparison to the ideal model (**Figure 3D**).

### Higher or lower LIGHT expression is linked to different genomic alterations

We next explored the correlation between LIGHT expression and genomic change characteristics. When we investigated the somatic mutation profile data of TCGA, we found a higher mutation frequency of IDH1 (73%), TP53 (47%) and ATRX (33%) in the LIGHT^low^ group (**Figure S3A**, n = 352). In the LIGHT^high^ group, EGFR (19%), TTN (17%) and PTEN (17%) were identified with a higher mutation frequency (**Figure S3A**, n = 320). The expression of LIGHT was similarly positively correlated with somatic mutations (**Figure S4A**, slope = 0.023092, P = 4.011e-07). The TCGA dataset was then used for copy number variation analysis, and we found that amplification in chr7 and deletion in chr10 were both enriched in the LIGHT^high^ samples. On the other hand, the LIGHT^low^ samples showed a higher frequency of deletion in chr1 as well as chr19, which was a widespread genomic alteration among oligodendrogliomas (**Figure S3B**). LIGHT expression was also found to positively correlate with copy number alterations (CNAs) (**Figure S4B**, slope = 3.6238, P = 0.028595). We next employed GSITIC analysis to clarify the details of the chromosome changes, and we found 23 amplification and 34 deletion events (**Figure S3C**). In the LIGHT^high^ group, we discovered amplification peaks at a number of well-known oncogenic drivers, including EGFR (7p11.2), PDGFRA (4q12), and CDK4 (12q14.1). Conversely, we discovered deletion peaks at several tumor suppressor genes, including CDKN2A/CDKN2B (9p21.3) and PTEN (10q23.3). Moreover, separate pathways, such as the TCA cycle and glutathione metabolism, were affected by diverse chromosomal alterations according to LIGHT expression (**Figure S4C**).

### Analysis of LIGHT-related biological functions suggests that genes exhibiting positive correlations with LIGHT expression are enriched in immune processes

**Figure 4:**
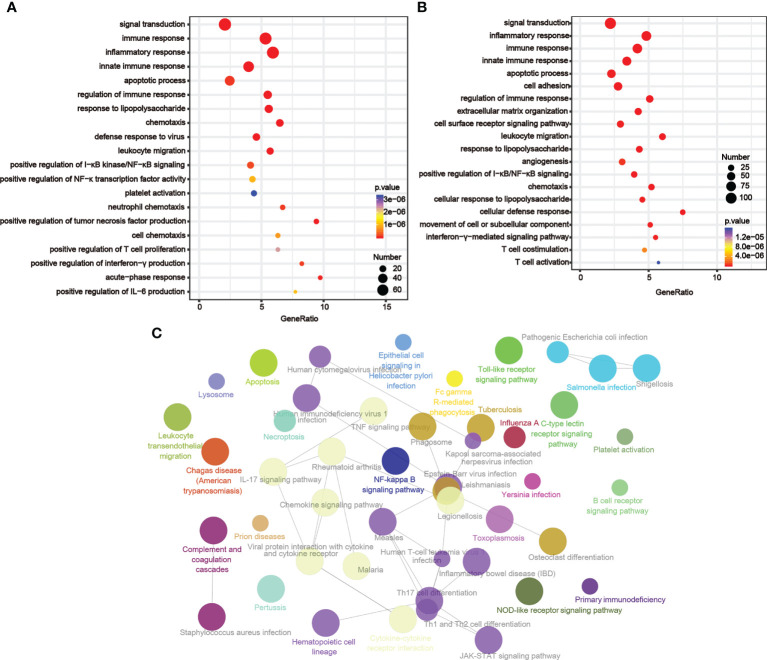
LIGHT-related biological functions in gliomas. Based on GO databases, the biological pathways shown in the graphs were identified by assessing the sets of LIGHT-associated genes in the **(A)** TCGA and **(B)** CGGA datasets. **(C)** Network graph exhibiting pathway terms enriched in LIGHT-positively-associated gene signatures.

By examining the genes whose expression levels had positive relationships with LIGHT expression (TCGA, n = 800; CGGA, n = 656; **Table S3**), we then used GO analysis to investigate the underlying function of LIGHT in gliomas. According to our findings, these genes were enriched in immune-related signaling pathways or processes, such as leukocyte migration, innate immune response, immune response regulation, inflammation response, immunological response, and immune response (**Figures 4A, B**). We next used KEGG pathway analysis to create a signaling network of these positively correlated genes, and the results demonstrated that LIGHT-related genes were extensively involved in immunological processes such as the NF-B signaling pathway, necroptosis, and apoptosis (**Figure 4C**). These data suggested that LIGHT might play essential role in the immunologic biological function of GBM.

### LIGHT participates in T-cell activities and inflammatory processes

LIGHT has been in preclinical development for over a decade and is believed to play a crucial role in immunotherapy (21, 38). We used GSEA to investigate the part LIGHT plays in T-cell activity (**Figure S5A**). The findings demonstrated a negative correlation between LIGHT and the control of T-cell mediated immunity, the control of the T-cell receptor signaling pathway, the control of T-cell activation, and the control of T-cell proliferation. The positively linked pathways, on the other hand, included T-cell apoptosis, T-cell differentiation, T-cell migration, T helper 1 type immune response, T helper cell differentiation, and so on. These findings showed that LIGHT suppressed T-cell activity, suggesting that LIGHT may help to reduce T-cell-associated antitumor immunity in the glioma microenvironment.

**Figure 5:**
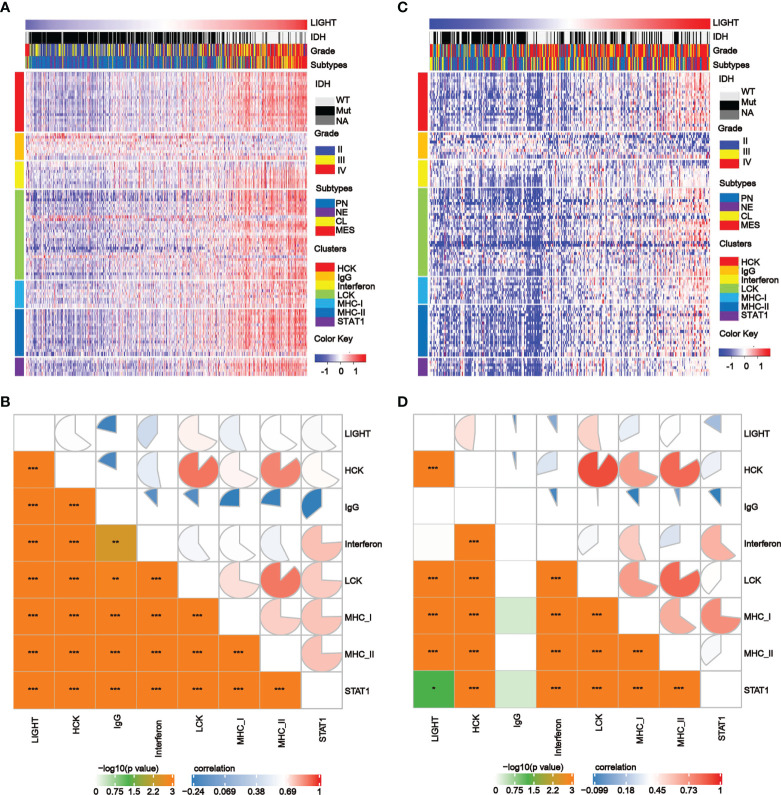
The correlation between LIGHT expression and T-cell immunity and immunity processes in all gliomas. **(A, C)** Heatmaps showing LIGHT-related immune activities of gliomas in the TCGA **(A)** and CGGA **(C)** datasets. The distribution of the LIGHThi and LIGHTlo groups, IDH status, WHO grade and molecular subtypes are displayed on the top of the heatmap, and the clusters of different immune activities are displayed on the left side. **(B, D)** Corrgrams illustrating the r values of Pearson correlation analysis between LIGHT and immunity metagenes in TCGA **(B)** and CGGA **(D)** datasets.

We further analyzed the correlations between LIGHT expression and inflammatory processes as previously reported (39). We discovered that LIGHT expression exhibited a positive correlation with HCK, interferon, LCK, MHC-I, MHC-II, and STAT1 metagenes in all gliomas, while IgG exhibited a negative correlation with LIGHT expression based on the TCGA and CGGA datasets. Based on the literature describing common inflammatory signatures, we found that LIGHT expression was positively correlated with HCK, interferon, LCK, MHC-I (**Figures 5A** **–D**). The analysis of GBM yielded comparable outcomes (**Figures S5B, C**).

### There is a widespread association between LIGHT expression and tumor microenvironmental immune or stromal cells

**Figure 6:**
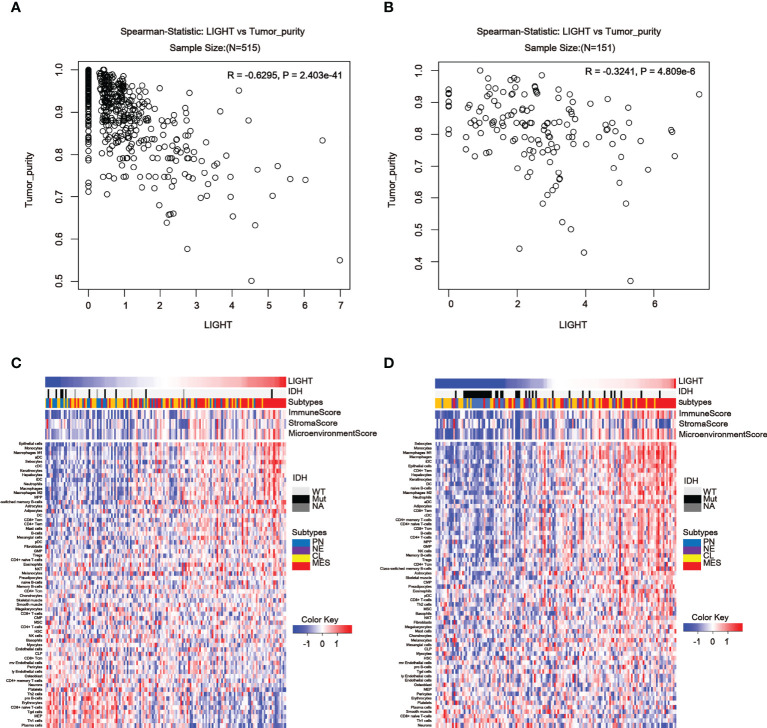
LIGHT in the tumor microenvironment is associated with tumor purity and immune or stromal cell populations. **(A, B)** In the TCGA **(A)** and CGGA datasets, LIGHT expression is negatively linked with tumor purity **(B)**. Heatmaps using TCGA **(C)** and CGGA GBM data in **(C, D)** show the relationship between LIGHT and 64 immune or stromal cell types **(D)**.

To further assess the link between LIGHT expression and immune functions, we estimated the correlations between LIGHT expression and immune cell populations in the tumor microenvironment. In the TCGA dataset, we found an obviously negative correlation between LIGHT expression and tumor purity in both LGG (**Figure 6A**, R = -0.6295, P = 2.403e-41) and GBM (**Figure 6B**, R = -0.3241, P = 4.809e-6), indicating that LIGHT expression might increase the complexity of gliomas. To explore whether this enhanced complexity by LIGHT expression was caused by immune cells, we analyzed the correlation between LIGHT expression and 64 immune/stromal cell subtypes (15). LIGHT expression and immunological score as well as microenvironment score were correlated (**Figures 6C, D**). More specifically, the thorough analysis of LIGHT’s roles in tumor-cell interactions suggested a positive relationship between LIGHT expression and infiltrating cells like monocytes, M1 macrophages, neutrophils, DCs, CD8+ T effector memory cells (TEM), CD4+ TEM cells, and NK cells, while a negative relationship was found in pro-B cells, CD8 naive T cells, Th1 cells, and plasma cells. Together, these findings suggest that increased LIGHT expression may make it easier for immune and stromal cells to enter the tumor microenvironment.

### Correlation between immune checkpoint molecules and LIGHT expression

**Figure 7:**
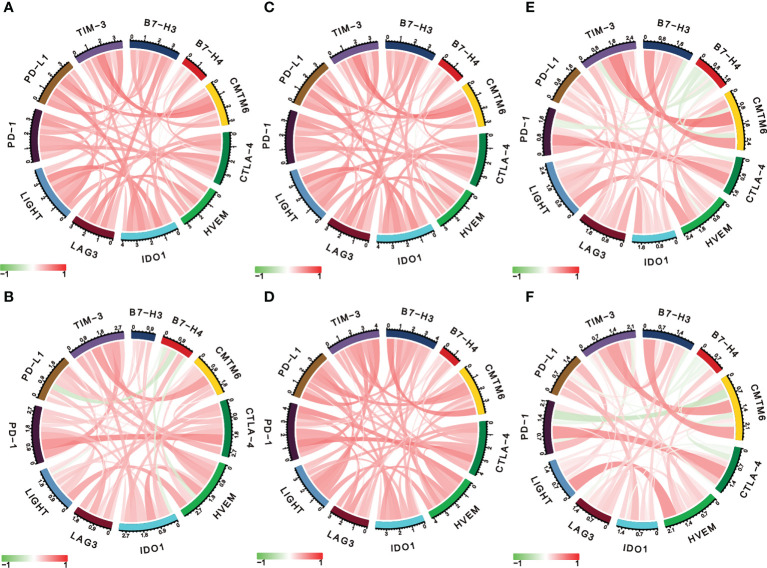
LIGHT is correlated with other immune checkpoint molecules in gliomas. Correlation analysis of LIGHT and several immune checkpoint molecules in LGGs (upper row) and GBMs (lower row) in the TCGA **(A, B)**, CGGA **(C, D)**, and Rembrandt datasets **(E, F)**.

Considering the essential roles of checkpoint molecules in immune processes, we conducted analyses on the correlations between LIGHT expression and well-known immune checkpoint molecules. Thus, in LGG, we found a significant correlation between LIGHT and immunological checkpoint proteins such as IDO1, CTLA-4, PD-1, PD-L1, and TIM-3 (**Figure 7A, C** **, E**), whereas in GBM, we found a significant correlation between PD-1, PD-L1, CTLA-4, and B7-H3 (**Figures 7B, D** **, F**). In LGG and GBM from the TCGA, CGGA, and Rembrandt datasets, the association between its receptor HVEM, which was also shown to have a significant role in immunological processes, was evident (**Figures 7A** **–F**). These findings further support LIGHT’s critical role in immunity by showing a broad connection between LIGHT and immunological checkpoint genes.

### Mutation features of LIGHT in the TCGA database

**Figure 8:**
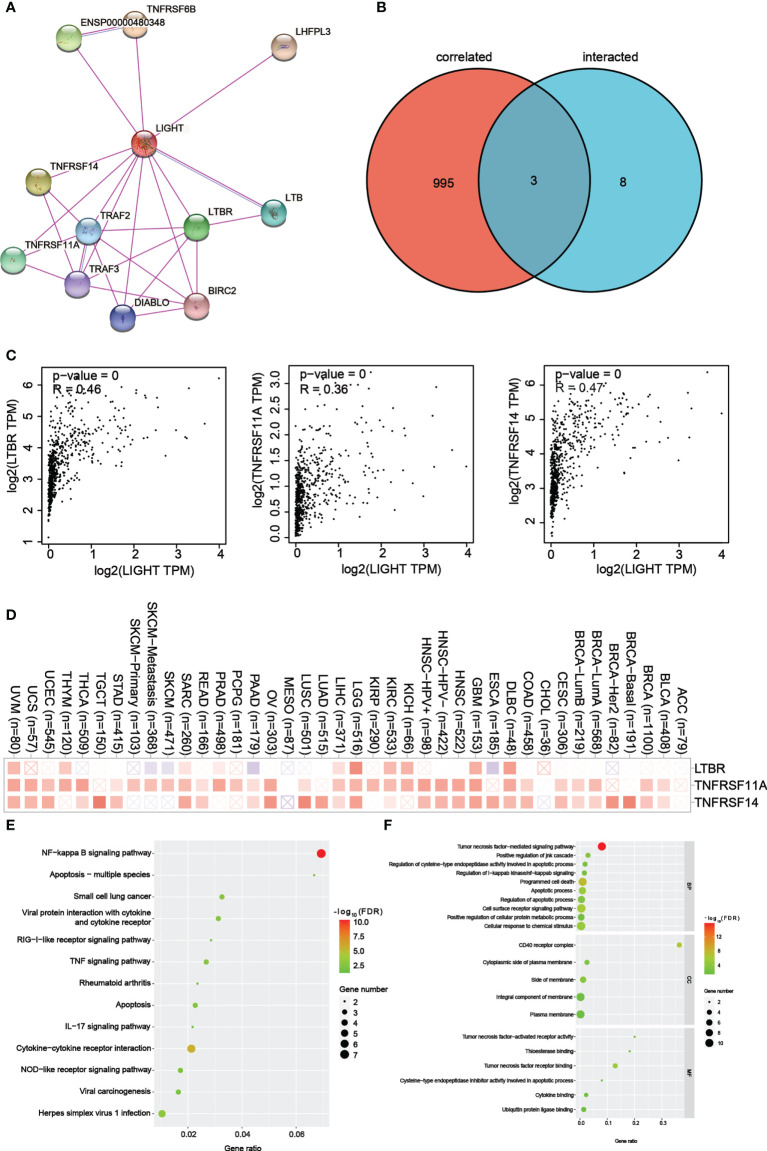
LIGHT-related gene enrichment analysis. **(A)** We first obtained the available experimentally determined LIGHT-binding proteins using the STRING tool. **(B)** An intersection analysis of LIGHT binding (from STRING) and correlated genes (from GEPIA2) was conducted. **(C)** Expression correlation between LIGHT and selected targeting genes, including LTBR, TNFRSF11A and TNFRSF14. **(D)** The corresponding heatmap data in the detailed cancer types are displayed. **(E)** Based on the LIGHT-binding proteins, KEGG pathway analysis was performed. **(F)** (The molecular function data in GO analysis are also shown.

**Figure 9:**
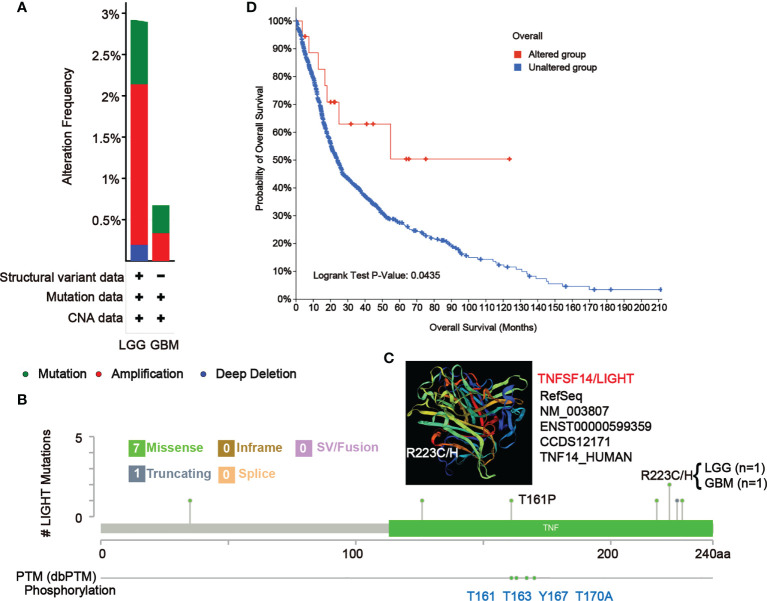
Mutation features of LIGHT in different tumors of TCGA. The alteration frequency of mutation type **(A)** and mutation site **(B)** in GBM and LGG samples are showed. We display the mutation site with the highest alteration frequency (R223C/H) in the 3D structure of LIGHT **(C)**. **(D)** We also analyzed the potential correlation between mutation status and the overall survival of GBM and LGG patients using the cBioPortal tool.

In LGG and GBM samples from the TCGA cohort, we looked at the genetic modification status of LIGHT. The patients with LGG with “amplification” as the primary type had the highest-frequency alteration of LIGHT (> 2%), as shown in **Figure 8A**. With an alteration frequency of 0.35%, the “amplification” type and “mutation” type both displayed equal values in GBM instances (**Figure 9A**). Further presented in **Figure 8B** are the types, locations, and case numbers of the LIGHT genetic alterations. R223C/H change was reported in one case of LGG and one case of GBM, and missense mutation of LIGHT was discovered to be the most common type of genetic alteration (**Figure 9B**), inducing a frameshift mutation of the LIGHT gene that switched residue 223 of the protein from R (arginine) to C (cystine) or H (histidine), followed by LIGHT protein truncation. We observed the R223 site in the 3D structure of the LIGHT protein (**Figure 9C**). Additionally, we found that T161, as one of four phosphorylation sites of LIGHT, can be mutated to P (proline). The T161P mutation could block the phosphorylation of LIGHT and possibly influence its function (**Figure 9B**). Additionally, we explored the potential association between genetic alterations in LIGHT and the clinical survival prognosis of cases. The data in **Figure 9D** indicate that LGG and GBM cases with altered LIGHT showed better prognosis overall (P = 0.0435) than cases without LIGHT alteration.

### LIGHT-binding proteins and their function in tumor progression

For a series of pathway enrichment analyses, we tried to weed out LIGHT-binding proteins and genes whose expression is linked with LIGHT in order to better understand the molecular mechanism underlying the LIGHT gene’s role in the development of cancer. Using the STRING tool, we discovered 11 LIGHT-binding proteins, all of which had experimental backing. **Figure 8A** shows the protein interactions in this network. Using the GEPIA2 tool, we aggregated all TCGA tumor expression data to identify the top 998 genes that linked with LIGHT expression. Two datasets were intersected to produce three genes (**Figure 8B**). There was a strong correlation between the expression levels of LTBR, TNFRSF11A, and TNFRSF14, as can be seen in **Figure 8C**. The corresponding heatmap data also showed a positive correlation between LIGHT and the aforementioned 3 genes in the majority of the specific cancer types (**Figure 8D**).

To conduct KEGG and GO enrichment analyses, datasets analyzed with LIGHT-binding genes. According to the KEGG information in **Figure 8E**, “NF-kappa B signaling pathway” and “Apoptosis multiple species” may be involved in how light affects tumor pathogenesis. The results of the GO enrichment study also showed that the majority of these genes were associated with the “CD40 receptor complex” and “tumor necrosis factor-mediated signaling pathway.” (**Figure 8F**).

### Role of LIGHT expression in response to ICB therapy

**Figure 10:**
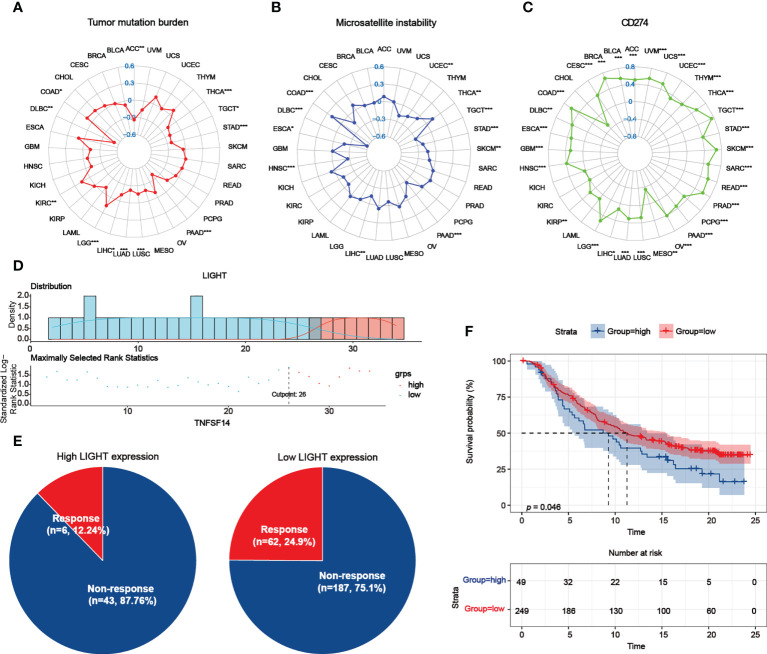
Role of LIGHT expression in response to ICB therapy. **(A–C)** The association between LIGHT expression and TMB **(A)**, MSI **(B)**, and CD274 expression levels **(C)** was shown by radar graphs. The correlation coefficient is shown by the dots in radar charts. **(D)** The “survminer” package’s optimal cutoff values for the low- or high-LIGHT expression group. **(E)** The percentage of patients with low or high LIGHT expression that responded to PD-L1 block immunotherapy was depicted in pie plots. **(F)** The Kaplan-Meier curve in the anti-PD-L1 immunotherapy cohort indicated the survival of the patient groups with high and low LIGHT expression (IMvigor210 dataset; P = 0.046, Log-rank test).

The tumor mutational burden (TMB) and LIGHT expression were positively correlated in COAD, KIRC, and LGG but negatively correlated in ACC, DLBC, LIHC, LUAD, LUSC, PAAD, STAD, TGCT, and THCA (**Figure 10A**). Microsatellite instability (MSI) was found to be correlated with COAD and THCA, as well as with DLBC, ESCA, HNSC, LIHC, PAAD, SKCM, STAD, TGCT, and UCEC (**Figure 10B**). Additionally, **Figure 10C** demonstrates that LIGHT expression and CD274 have a positive correlation in the majority of cancer types, but MESO has a negative correlation. As an extra degree of verification, the clinical immunotherapy cohort IMvigor210 was employed. Anti-PD-L1 blockers had a range of effects on the patients in the IMvigor210 cohort, including complete response (CR), partial response (PR), stable disease (SD), and progressive disease (PD). We determined the optimal cutoff value and split the samples into groups with high and low LIGHT expression levels taking into account LIGHT expression levels and connected prognostic circumstances (**Figure 10D**). We found that the response ratio in the groups with low light expression was much higher than the response ratio in the groups with high light expression (**Figure 10E**). A worse prognosis was also indicated by greater expression of LIGHT (**Figure 10F**).

## Discussion

In this study, we comprehensively employed large-scale in silico analyses on LIGHT with molecular and clinical datasets extracted from TCGA, CGGA and Rembrandt. First, we confirmed the expression characteristics of LIGHT, in which gliomas showed higher LIGHT expression than normal tissues, and higher LIGHT expression was observed in higher-grade gliomas. The mesenchymal subtype, whose prognosis is widely accepted as the worst among gliomas, manifested the highest LIGHT expression among the four subtypes classified by molecular signatures. Higher LIGHT expression sensitively predicted a worse prognosis in glioma patients. In addition, we found several deletions and amplifications of chromosomal regions or genes in LIGHThigh or LIGHTlow clusters. Furthermore, we analyzed the function of LIGHT in immune processes. The results exhibited a tight correlation between immune actors such as HCK and interferon, and several immune and stromal cell populations were shown to be linked with LIGHT expression. LIGHT was proven to have a widespread correlation with several immune checkpoint proteins, such as PD-1, PD-L1 and CTLA-4. These results suggest an essential role for LIGHT in immunity. To translate our results into practical application, we developed a nomogram model applying LIGHT expression level and several risk factors, and the nomogram model’s efficacy was identified by a calibration plot.

Immunosuppression in gliomas, especially in GBM, has been widely noticed, and GBM is regarded as an immune evader among cancers (8, 9). Immune therapy for GBM is urgently needed by GBM patients. The classical immunosuppression mechanisms in cancers include the infiltration of immunosuppressive cells and the expression of inhibitory immune checkpoint molecules (ICMs). The infiltrating immunosuppressive immune cells mainly include myeloid-derived suppressor cells (MDSCs), M2-type macrophages (M2Mφs), regulatory T cells (Tregs) and regulatory B cells (Bregs) (40), while several inhibitory ICMs expressed in the glioma microenvironment have been reported: PD-L1 (10), galectin-1 (41), galectin-9 (42), and HVEM (15). Tregs are known to play essential roles in the glioma microenvironment. LIGHT was reported to facilitate the expansion of Tregs without reducing their inhibitory functions (43), and another study observed increased CD8+ T-cell infiltration in a murine model of prostate cancer in which LIGHT-expressing cancer cells were intraprostatically injected (44). This was confirmed by our results that LIGHT functioned as a T-cell activity suppressor. However, they also observed that highly expressed LIGHT could inhibit the function of Tregs (43), and forced LIGHT expression in murine prostate tumor models also inhibited Tregs and cancer progression (19). Therefore, there is a double-edge effect of LIGHT in Tregs, and its function requires further research. On the other hand, to date, several immunotherapies for GBM targeting inhibitory ICMs have been established and named immune checkpoint inhibitors (ICIs). Previous studies found that a monoclonal antibody targeting CTLA-4 (9H10) could reverse immune evasion in well-established murine glioma models (45). Given that TIM-3 and LAG-3 are expressed in tumor-infiltrating lymphocytes functioning as coinhibitory receptors that could induce immune exhaustion in the microenvironment of GBM (46, 47), researchers applied a combination of anti-TIM3 + anti-PD1 + focal radiation as well as anti-LAG3 + anti-PD1 in murine models, and the survival was significantly prolonged (48, 49). Unfortunately, despite the favorable results in preclinical models, the efficacy of the ICIs in most clinical trials was shown to be limited (50, 51). Here, we report the positive linkage of LIGHT with ICMs, such as IDO1, CTLA-4, PD-1, PD-L1 and TIM-3, and provide an underlying therapeutic target for the development of new immunotherapies to aid the ICIs.

LIGHT is a 29 kD protein that is mainly expressed on immature dendritic cells, activated natural killer (NK) cells and activated T cells (22, 52), and its expression sites determine its main functions in immune processes. Previous studies reported that LIGHT could be involved in the establishment of an immune microenvironment breaking immune tolerance to self-antigens (53), and LIGHT expressed in the tumor microenvironment could lead to the infiltration of lymphocytic cells (29, 54). In our study, we also found a negative correlation between LIGHT expression and tumor purity, and a positive correlation between LIGHT expression and immunity metagenes, excluding IgG, such as HCK and interferon, was observed. In addition, its expression was found to be positively associated with the immune score and microenvironment score. These results are in line with the earlier studies.

According to previous studies, LIGHT has prognostic value in several cancers. Higher expression of LIGHT predicted more adverse clinicopathological features and a worse prognosis in renal cell carcinoma (55). An in silico study of GBM indicated that high expression of LIGHT, together with IDO1, predicted a lower overall survival rate (35). A study of melanoma found an 8-gene local gene signature and established a risk prediction model with an equation. In this equation, LIGHT had the greatest parameter among the genes positively contributing to the risk score, which means that higher LIGHT expression was the most potent index to predict a higher risk of melanoma (56). Similarly, in our results, the highest LIGHT expression was found in the MES subtype, which was considered to show the worst expression, and higher LIGHT expression could also predict worse overall survival in gliomas, including GBM and LGG. The nomogram model established based on LIGHT expression and other risk factors also exhibited an acceptable prognostic capability.

Taken together, our study reveals a potential role for LIGHT in gliomas as a prognostic marker and an underlying therapeutic target for immunotherapy. The detailed functions and mechanisms of LIGHT in glioma and its therapy warrant further research.

## Data availability statement

The original contributions presented in the study are included in the article/**Supplementary Material**. Further inquiries can be directed to the corresponding author.

## Ethics statement

This study was reviewed and approved by the Research Ethics Committee of Shandong University and the Ethics Committee of Qilu Hospital (Shandong, China) (SDULCLL2021-2-26). Written informed consent was obtained from the individual(s) for the publication of any potentially identifiable images or data included in this article.

## Author contributions

YS, MH, and WeiZ designed the project and wrote the paper; YS, MH, WenZ, and ZX performed the in vitro experiments. ZX, GX, and ZJ collected patient samples; MH, XW, and YS performed statistical analysis. All authors contributed to the article and approved the submitted version.

## Funding

This work was supported by the Research Project of Jinan Microecological Biomedicine Shandong Laboratory (JNL-2022041C), Shandong Excellent Young Scientists Fund Program (2022HWYQ-035), Shandong Provincial Natural Science Foundation (ZR2021QH030), the Qilu Young Scholar Program of Shandong University, the 2021 Shandong Medical Association Clinical Research Fund (YXH2022ZX02194). Qilu Special Project (YXH2022ZX02194), the Special Foundation for Taishan Scholars.

## Conflict of interest

The authors declare that the research was conducted in the absence of any commercial or financial relationships that could be construed as a potential conflict of interest.

## Publisher’s note

All claims expressed in this article are solely those of the authors and do not necessarily represent those of their affiliated organizations, or those of the publisher, the editors and the reviewers. Any product that may be evaluated in this article, or claim that may be made by its manufacturer, is not guaranteed or endorsed by the publisher.
