# Supplementary material for: Comprehensive characterization of TNFSF14/LIGHT with implications in prognosis and immunotherapy of human gliomas
Source: Front Immunol. 2022 Oct 20;13:1025286. doi: 10.3389/fimmu.2022.1025286 (PMC9632349; doi:10.3389/fimmu.2022.1025286)
Supplement: Supplementary file 1 [file Table_1.docx]

**Table S1** Univariate and multivariate Cox regression of *LIGHT* expression for overall survival in TCGA and CGGA glioma patients

CGGA

| Variable | Univariate Cox Regression | | Multivariate Cox Regression | |
| --- | --- | --- | --- | --- |
|  | HR (95 % CI) | *P* | HR (95 % CI) | *P* |
| Age  Increasing years | 1.026 (1.018-1.035) | <0.001 | 1.009 (1.001-1.017) | 0.024 |
| Gender  Female *vs* male | 0.946 (0.775-1.154) | 0.585 |  |  |
| WHO grade  High- *vs* low- | 3.727 (3.043-4.566) | <0.001 | 1.571 (1.390-1.776) | <0.001 |
| *LIGHT* expression  High *vs* low | **1.125 (1.089-1.161)** | **<0.001** | **1.056 (1.012-1.102)** | **0.012** |
| *IDH* status  Mutation *vs* wild-type | 0.244 (0.170-0.350) | <0.001 | 0.638 (0.465-0.876) | 0.005 |
| Radiotherapy  Yes *vs* no | 0.833 (0.645-1.075) | 0.160 |  |  |
| Chemotherapy  Yes *vs* no | 0.800 (0.627-1.021) | 0.073 |  |  |

TCGA cohort

| Variable | Univariate Cox Regression | | Multivariate Cox Regression | |
| --- | --- | --- | --- | --- |
|  | HR (95 % CI) | *P* | HR (95 % CI) | *P* |
| Age  Increasing years | 1.069 (1.058-1.080) | <0.001 | 1.034 (1.021-1.047) | <0.001 |
| Gender  Female *vs* male | 1.119 (0.680-1.842) | 0.657 |  |  |
| WHO grade  GBM *vs* LGG | 8.967 (6.795-11.832) | <0.001 | 1.967 (1.325-2.920) | <0.001 |
| *LIGHT* expression  High *vs* low | **1.530 (1.437-1.629)** | **<0.001** | **1.107 (1.009-1.215)** | **0.031** |
| *IDH* status  Mutation *vs* wild-type | 0.101 (0.076-0.135) | <0.001 | 0.319 (0.202-0.506) | <0.001 |
| 1p/19q status  Codel *vs* Non-codel | 0.217 (0.137-0.344) | <0.001 | 0.547 (0.315-0.949) | 0.032 |

HR, hazards ratio; CI, confidence interval.
