# Supplementary material for: Comprehensive characterization of TNFSF14/LIGHT with implications in prognosis and immunotherapy of human gliomas
Source: Front Immunol. 2022 Oct 20;13:1025286. doi: 10.3389/fimmu.2022.1025286 (PMC9632349; doi:10.3389/fimmu.2022.1025286)
Supplement: Supplementary file 4 [file DataSheet_1.docx]

Supplementary Material


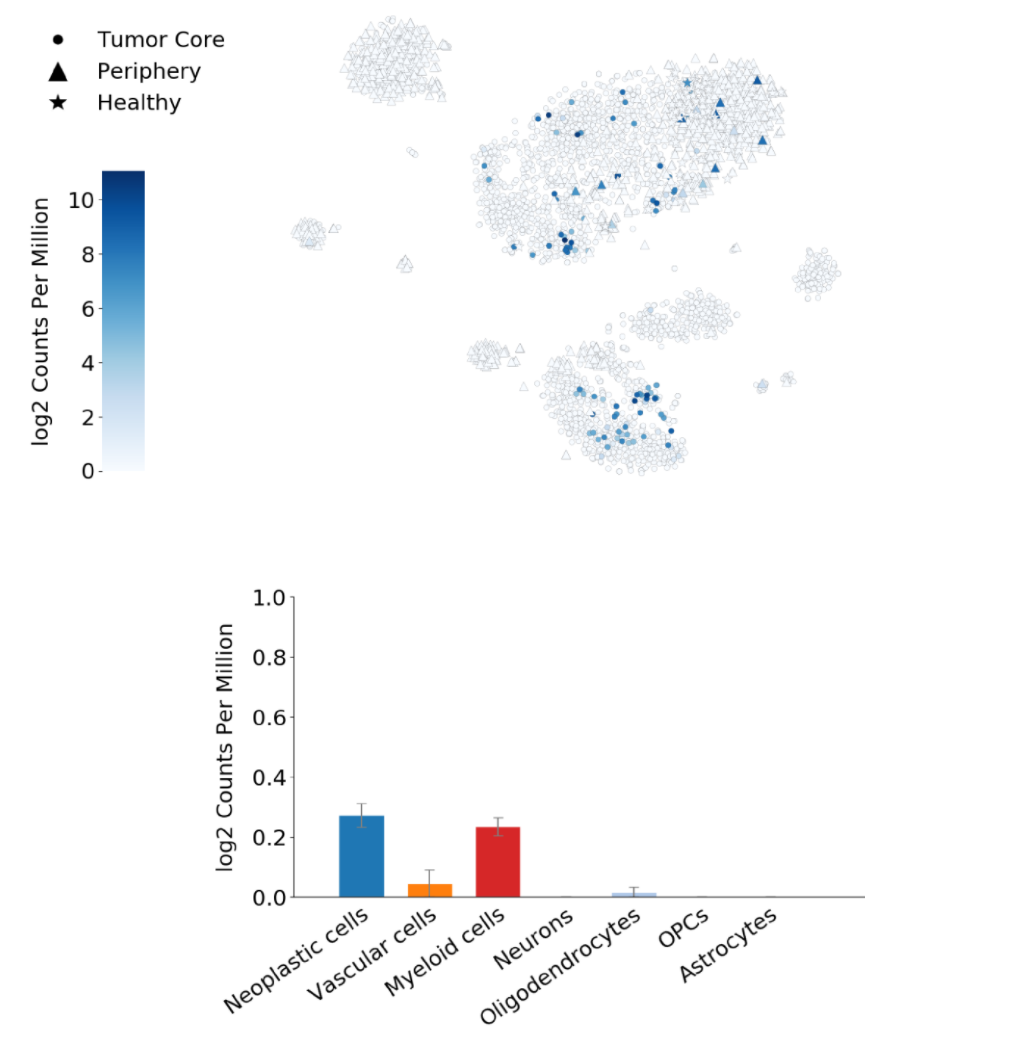

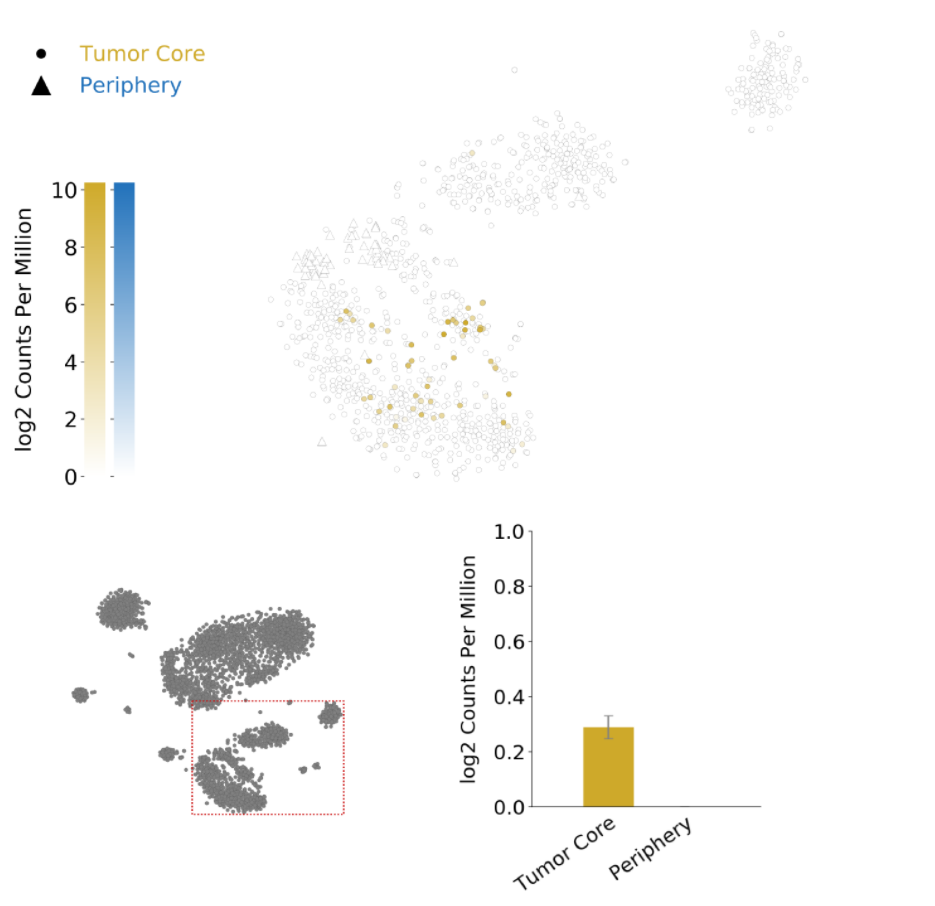


**A**

**B**

**Supplementary Figure 1.** Single-cell sequencing showing the expression patterns of LIGHTs. (A) Column plot illustrating LIGHT mainly expresses in neoplastic and immune cells. (B) Column plot illustrating LIGHT mainly expresses in tumor core rather than periphery regions.


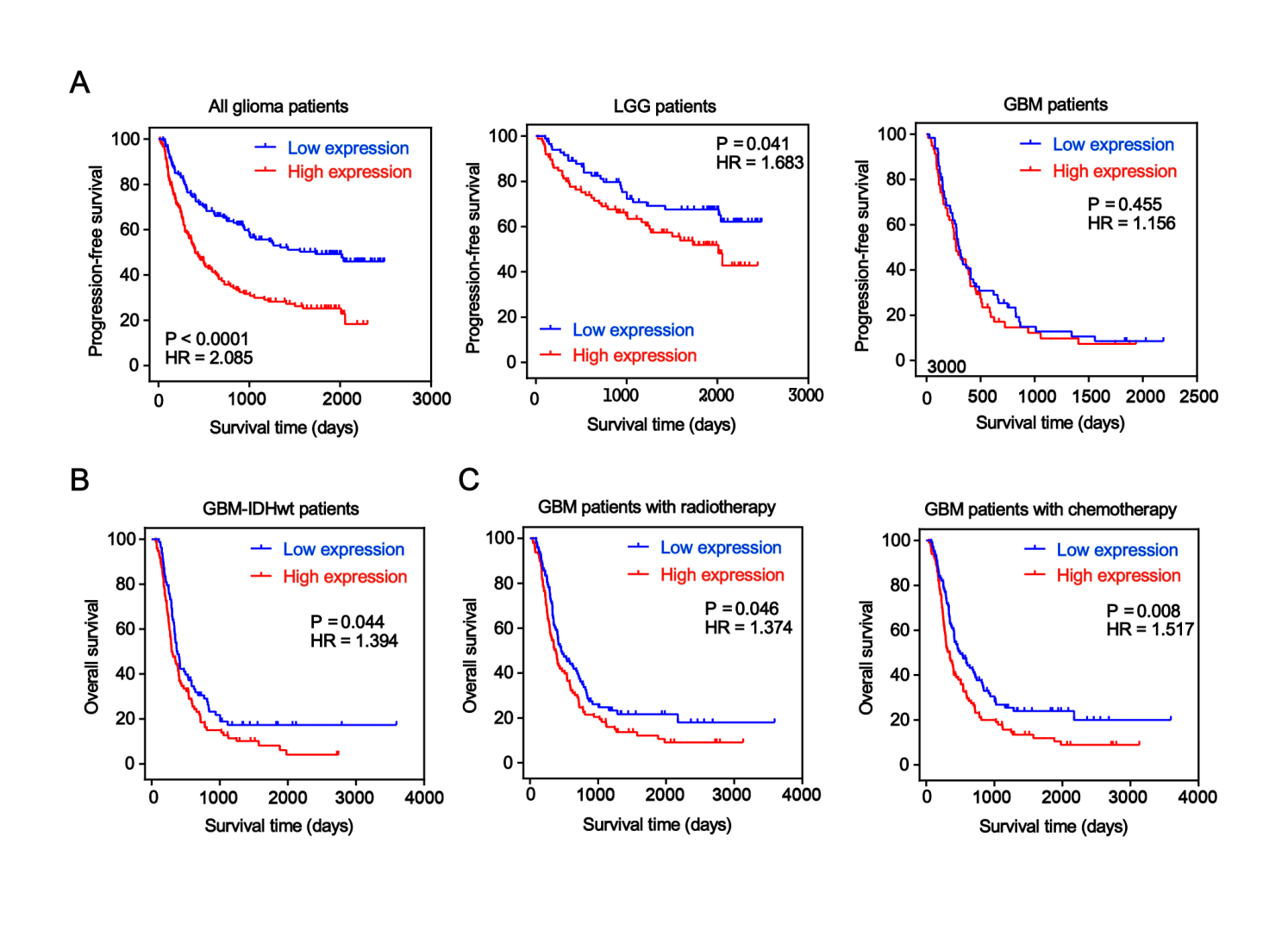


**Supplementary Figure 2.** The prognostic efficacy of LIGHT in progression-free period of gliomas, IDHwt GBM and post-therapy GBM analyzed by Kaplan-Meier curves. (A) A sensitive prognostic effect of LIGHT in progression-free survival (PFS) rate of all glioma and LGG patients is observed that higher LIGHT expression predicts poorer prognosis. The prognostic effect of LIGHT is not observed in GBM patients. (B) A more unfavorable overall survival (OS) rate of IDHwt GBM is predicted by higher LIGHT expression. (C) A more dismal prognosis of OS can be predicted by higher expression of LIGHT.


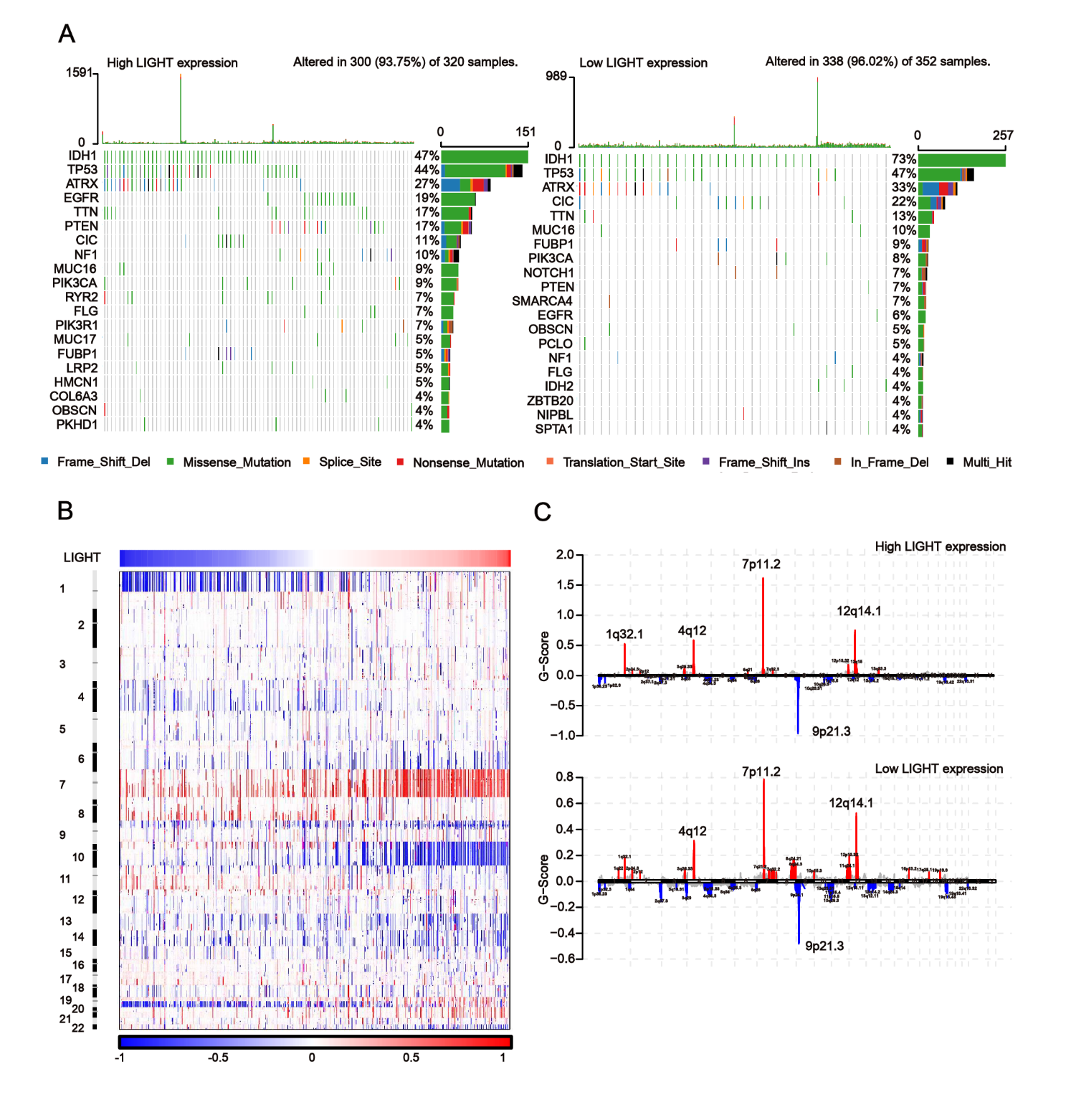


**Supplementary Figure 3. The linkage between genomic alterations and low or low LIGHThigh expression.** (A) Spectrum plot of somatic mutations in the LIGHThi and LIGHTlo groups. (B) Heatmap showing the copy number variation (CNV) in all the data divided by LIGHThi vs. LIGHTlo. Red represents amplification, and blue represents deletion. (C) Detailed frequency of chromosome changes in the LIGHThi and LIGHTlo groups. The X-axis represents the regions of chromosomes, and the Y-axis represents the G score, which indicates the frequency of chromosomal deletion (marked in blue) or amplification (marked in red).


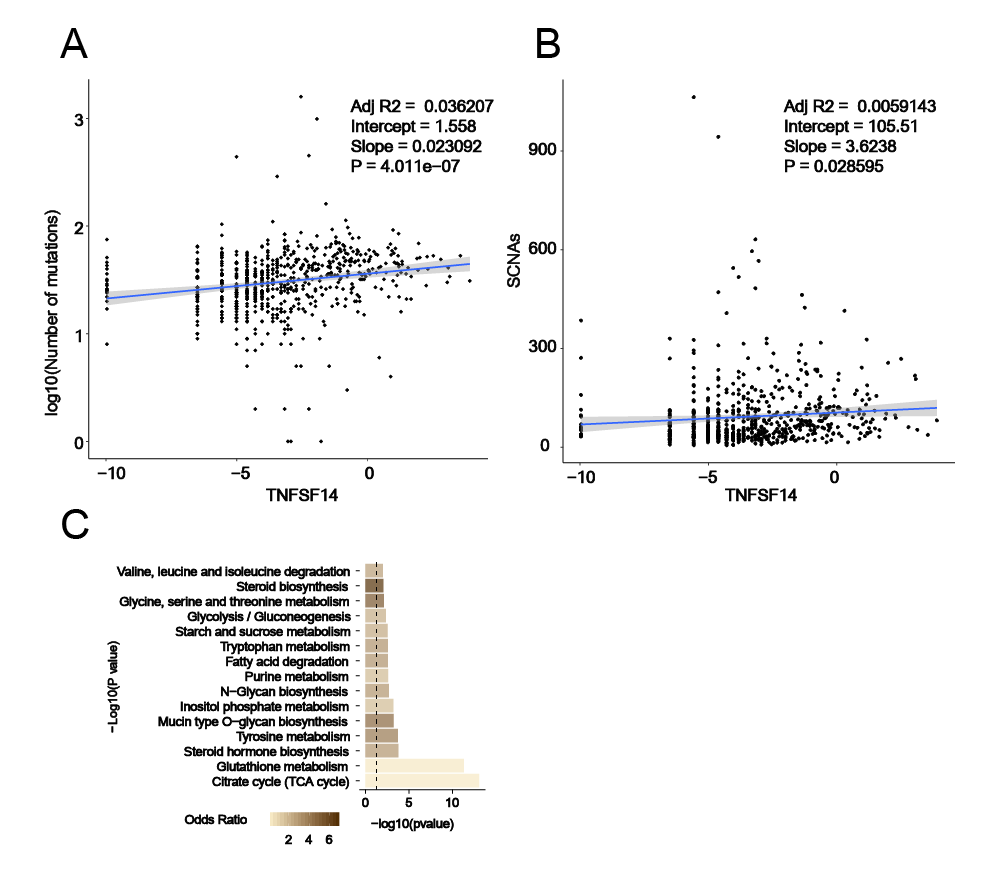


**Supplementary Figure 4.** Correlation between LIGHT expression and somatic mutations and CNAs. High LIGHT expression predicted more somatic mutations (A) and CNAs (B). (C) Pathways involved in differential genomic mutations according to LIGHT expression.


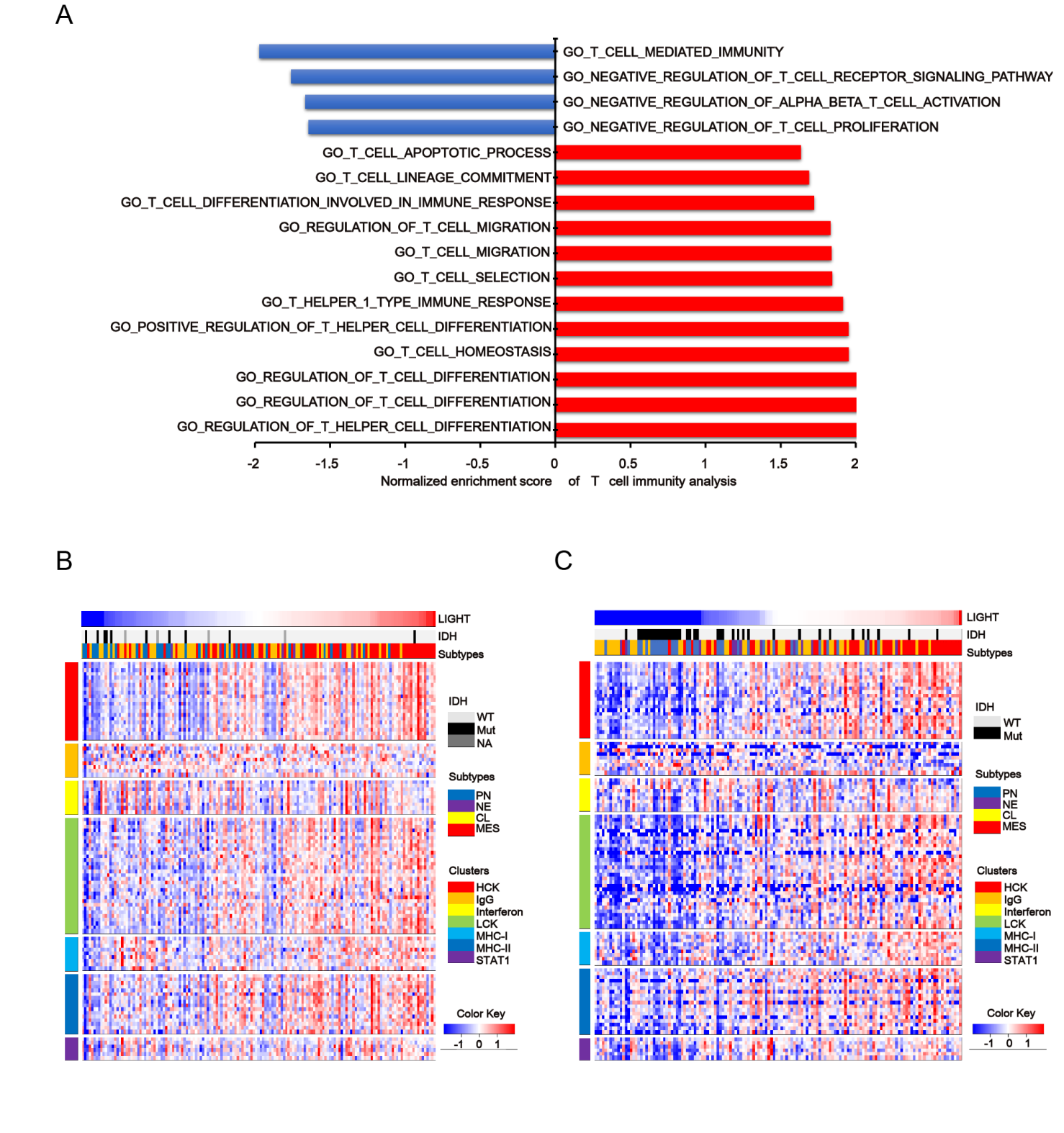


**Supplementary Figure 5.** The correlation between LIGHT expression and T cell immunity and immunity processes in GBM. (A) GSEA analysis showing the correlation between LIGHThi vs lo gene signatures and T cell immunity. The X-axis displays the NES. Positive association is marked in blue while negative association is marked in red. (B and C) Heatmaps manifesting LIGHT-related immune activities of gliomas in TCGA (B) and CGGA (C) datasets. The distribution of LIGHThi and LIGHTlo group, IDH situations, WHO grade and molecular subtypes are displayed on the top of heatmap and the clusters of different immune activities are displayed on the left side.
